# Treatment of Azo Dye Wastewater by Anaerobic Membrane Bioreactors: A Review on Process Performance and Mitigation Strategies

**DOI:** 10.3390/membranes16100334

**Published:** 2026-10-07

**Authors:** Jia-Ning Chen, Pengcheng Liu, Hao Wu, Xiang Li, Zhe Kong, Xueying Zhang, Yong Hu

**Affiliations:** 1 School of Environmental Science and Engineering, Nanjing Tech University, Nanjing 211816, China; cjning717@163.com (J.-N.C.); wwwlpc2021@163.com (P.L.); wuhao10620028@163.com (H.W.); lixiang429612@163.com (X.L.); 2 College of Material Science and Engineering, Nanjing Tech University, Nanjing 211816, China; 3 School of Environmental Science and Engineering, Suzhou University of Science and Technology, Suzhou 215009, China; zhekong@mail.usts.edu.cn

**Keywords:** azo dye, anaerobic membrane bioreactor, membrane fouling, chemical structure, mitigation strategy

## Abstract

Membrane fouling and biological toxicity are two main bottlenecks in treating azo dye wastewater using anaerobic membrane bioreactors (AnMBRs). This review examines how azo dye molecular structure affects AnMBR performance and membrane fouling. It discusses azo dye reduction, aromatic amine formation, microbial inhibition, and foulant formation. Aromatic amines may inhibit methanogenesis and promote the accumulation of hydrophobic foulants on membrane surfaces. At low dye loadings, stable treatment may be supported under favorable operating conditions, whereas higher loadings may inhibit anaerobic activity and accelerate membrane fouling. The review evaluates membrane modification, biological enhancement and in situ electro-cleaning strategies. No single strategy has yet ensured long-term industrial operation. Reported concentration thresholds are mainly derived from single-dye laboratory studies and may not apply to actual textile wastewater. Future work should use data-driven fouling prediction and pilot trials with real wastewater to define AnMBR operating limits.

## 1. Introduction

The rapid growth of the global population has driven the rapid expansion of the textile industry [1]. Generally, wastewater produced during textile manufacturing accounts for approximately 54% of global dye wastewater discharge [2,3]. Specifically, textile enterprises utilize various dyes to color different raw materials, such as cellulose, protein, and synthetic fibers, through processes such as dyeing and printing, which can generate wastewater with a complex composition containing dyes, salts, and sizing agents [4,5,6]. The average concentration of dye in textile wastewater is approximately 300 mg/L, and even 50 mg/L is highly undesirable and environmentally hazardous [7,8].

Among various dye categories, azo dyes are the most abundant and are characterized by one or more nitrogen-nitrogen double bonds (-N=N-), known as azo groups. Azo dyes in wastewater can be removed by physical, chemical, or biological methods [9]. Azo groups are typically resistant to aerobic biodegradation, whereas under anaerobic conditions, they undergo a relatively simple reduction process, relying on cofactors and redox mediators to cleave the azo bond for dye decolorization [10]. Azo dye reduction can be mediated by biological processes, representing a combination of biotic and abiotic reactions [11]. Specifically, biotic reduction proceeds via enzyme- or enzyme-cofactor-mediated reactions, while abiotic reduction occurs through purely chemical reactions involving reductants such as sulfides, which can accelerate the overall reduction process. Although both biotic and abiotic reactions can occur under anaerobic conditions, Van der Zee et al. found that biotic reactions dominate the dye decolorization process [11]. In addition, Xu et al. emphasized that anaerobic microorganisms can cleave azo bonds via specific reductases, achieving dye decolorization while simultaneously converting organic matter into methane energy, which can significantly reduce treatment energy consumption [12].

The removal of azo dyes from wastewater can be accomplished with physical, chemical or biological processes. For example, Rapo et al. used untreated eggshell waste as an adsorbent for Remazol Brilliant Violet-5R (RBV-5R), achieving a maximum removal efficiency of approximately 95% under the optimized conditions [13]. Azo dyes themselves are not environmentally benign because of their persistence and potentially toxic transformation products. However, anaerobic biological treatment is a comparatively sustainable option because it avoids aeration and enables methane recovery [9,12]. Ozer Cinar et al. investigated the decolorization performance of RBV-5R by an anaerobic-aerobic sequencing batch reactor (SBR) and observed optimal decolorization performance and aromatic amine degradation at a cycle time of 24 h [14]. Furthermore, Carlos Eduardo Lach et al. employed bio-electro-Fenton technology for the treatment of the azo dye RBV-5R, achieving effective dye removal and decolorization [15]. Similarly, Inaba et al. evaluated the decolorization of 11 reactive dyes using anaerobic granular sludge obtained from an expanded granular sludge bed reactor (EGSB) pilot plant [16]. The EGSB was operated at 35–37 °C with a hydraulic retention time (HRT) of 15 h. Batch tests were conducted in 500 mL flasks containing 250 mL of model textile wastewater and 50 mL of granular sludge. The flasks were incubated under microaerobic conditions at 37 °C and 60 rpm for 7 d. Eight dyes achieved over 94% decolorization [16]. Table 1 compares decolorization reported for different biological reactor configurations. The results should not be interpreted as a direct ranking of reactor types because dye structure, influent composition, HRT, co-substrate availability, salinity, and operating conditions differ among studies. High decolorization alone does not demonstrate stable methanogenesis or low membrane-fouling potential. Although conventional anaerobic reactors can decolorize azo dyes, biomass washout at short HRTs may hinder the retention and acclimation of dye-reducing microorganisms and slow-growing methanogens. Anaerobic membrane bioreactors (AnMBRs) use membrane-based solid–liquid separation to decouple HRT from solids retention time, prevent biomass washout, and retain slow-growing microorganisms [17,18,19,20,21,22]. Yurtsever et al. compared an AnMBR and an aerobic membrane bioreactor (AeMBR) for the treatment of synthetic azo dye-containing textile wastewater [23]. Membrane retention of biomass increased the biomass concentration by approximately two- to three-fold. Both systems achieved COD removal efficiencies above 80%, whereas the AnMBR achieved nearly complete decolorization and the AeMBR achieved only partial color removal. This operational advantage is accompanied by a risk of membrane fouling. The accumulation of sludge particles, colloids, extracellular polymeric substances, and soluble microbial products can promote cake layer formation and pore blocking, thus decreasing permeate flux. Regular physical or chemical cleaning is therefore required to restore flux [21,22]. The addition of an optimized dose of powdered activated carbon can serve as a complementary fouling control strategy by adsorbing dissolved foulants, modifying sludge properties and reducing filtration resistance, thereby retarding transmembrane pressure (TMP) development and helping maintain stable membrane operation [24,25].

A number of reviews have laid the foundation for understanding the anaerobic treatment of textile wastewater and fouling control in AnMBR. Xu et al. summarized the application of anaerobic biological processes in the treatment of textile printing and dyeing wastewater, indicating that anaerobic microorganisms can achieve the decolorization of azo dyes through the redox cleavage mediated by nitrite reductase and convert organic matter into methane [12]. However, Xu et al. also pointed out the problem of aromatic amine accumulation, which may inhibit the methane-producing activity. AnMBRs face issues such as operation and membrane fouling when treating high-concentration wastewater. An et al. systematically reviewed fouling mechanisms in AnMBRs, emphasizing the need for a comprehensive pollution control strategy [21]. Zielińska and Ojo further evaluated the trade-off relationship between nutrient/energy recovery and pollution management in AnMBRs, arguing that physical and chemical cleaning is still indispensable [22]. Min et al. reviewed fouling control strategies including powdered activated carbon addition, membrane surface modification, and proposed that multiple strategies may be required for sustainable long-term operation [24]. Physical fouling control includes flux relaxation, air scouring, and appropriately designed backwashing to remove reversible cake layers. Optical coherence tomography has been used to monitor fouling layer changes during physical cleaning. In a gravity-driven membrane bioreactor, relaxation expanded the fouling layer, whereas air scouring applied at the end of relaxation enhanced biomass removal and flux recovery [37,38].

Xu et al. focused on anaerobic dye transformation, whereas An et al. and Min et al. mainly addressed general AnMBR fouling mechanisms and control strategies, respectively. In contrast, this review links azo dye molecular structure and aromatic amine formation with microbial inhibition, EPS/SMP production, and membrane fouling in AnMBRs. The following sections examine how azo bond number, substituents, and transformation products affect decolorization, methanogenesis, and foulant formation, and then evaluate the corresponding mitigation strategies.

## 2. Characteristics of Azo Dyes

**Figure 1:**
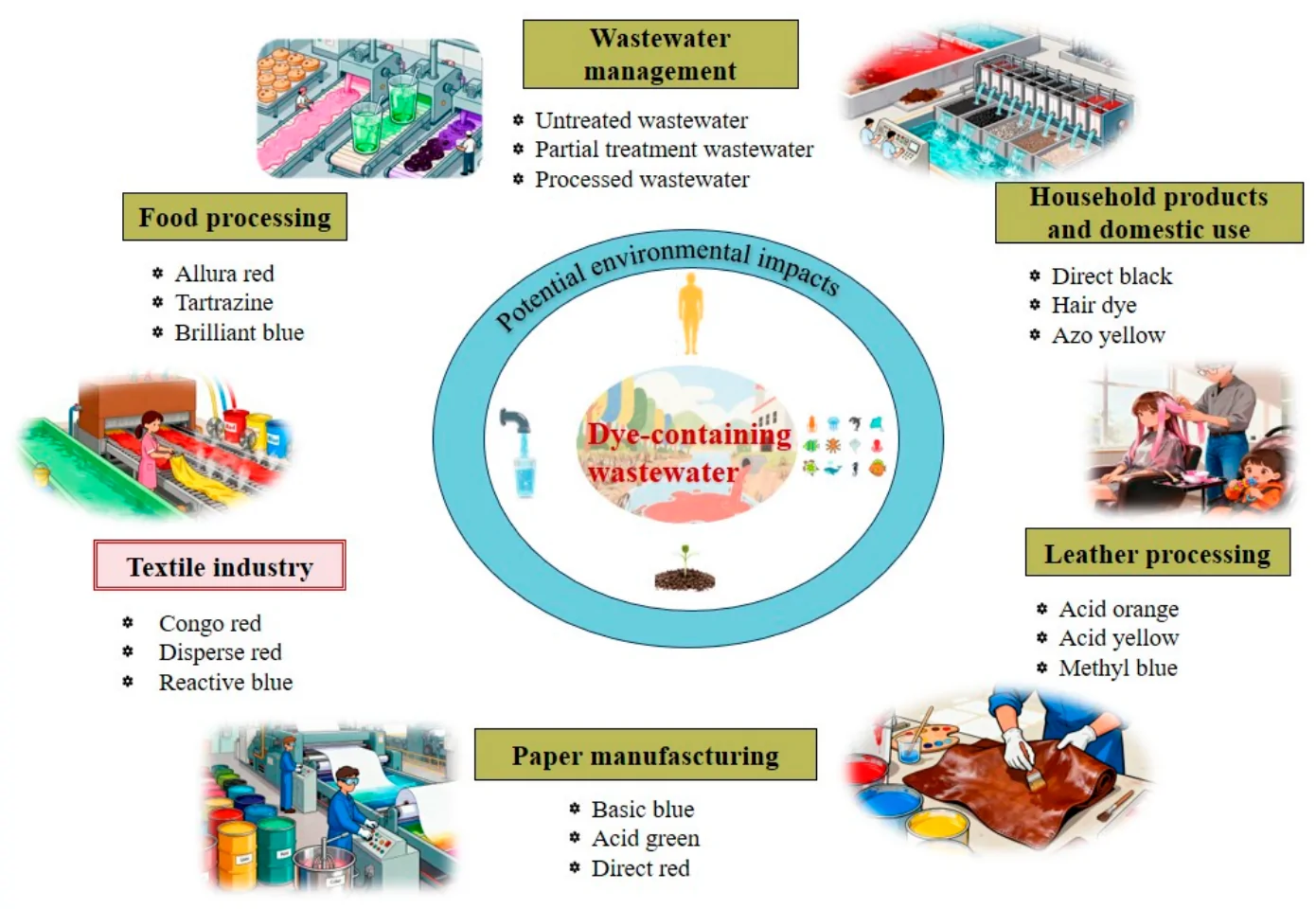
Major industrial and domestic sources of dye wastewater.

Dyes represent one of the most hazardous pollutants across various industries, including paint, food, textiles, papermaking, rubber, printing, and plastics. Chemically, dyes are organic compounds characterized by the structural integration of chromophores, auxochromes, and matrices [39,40]. Table 2 provides a structural comparison of representative synthetic dye classes. Based on their chromophore structures, dyes include azo, anthraquinone, indigo and sulfur dyes, etc. As illustrated in Figure 1, dye-containing wastewater can originate from textile dyeing, leather processing, paper manufacturing, food processing, and household product use.

### 2.1. Structures of Azo Dyes

As the primary pollutants in textile printing and dyeing wastewater, azo dyes possess molecular structural characteristics that directly influence their physicochemical properties. Azo dyes contain an azo bond (-N=N-), which serves as the chromophoric core and typically links two aromatic or heterocyclic rings [42].

Based on the number of azo bonds, azo dyes can be classified into monoazo, disazo, and polyazo dyes. An increase in the number of azo bonds generally leads to higher molecular weight and enhanced hydrophobicity, which can increase the difficulty of their biodegradation [52]. Most monoazo dyes feature a common linear conjugated chain of the type “-Ar-N=N-Ar’-”, with a relatively short conjugated system and simple spatial configuration, which makes them the most readily biodegradable dye type [53]. Under anaerobic conditions, the reduction efficiency of azo bonds is negatively correlated with the number of azo bonds, and azoreductases secreted by microorganisms can directly target and cleave the azo bond, achieving efficient reductive cleavage [12]. On the other hand, disazo and polyazo dyes form longer conjugated chain structures through the linkage of aromatic rings, and some even develop branched or cyclic conjugated systems, resulting in more stable molecular structures [54]. The cleavage of azo bonds requires multi-step reduction reactions, and the intermediate products (polycyclic aromatic amines) may exhibit higher toxicity and exert pronounced inhibitory effects on anaerobic microorganisms [55].

The substituents on the aromatic ring differ in polarity and electronegativity and can be broadly classified into hydrophilic and hydrophobic substituents. Hydrophilic substituents, represented by sulfonic acid groups (-SO_3_H) and hydroxyl groups (-OH), can enhance the water solubility of the dye, thereby facilitating contact with anaerobic microorganisms [56]. Yang et al. showed that the type and position of substituents can regulate bioelectrochemical decolorization kinetics by altering the electron density and steric hindrance around the azo bond. The effect of sulfonic acid substituents depended on their position relative to the azo linkage [57]. In contrast, hydrophobic substituents can enhance the stability of the azo bond, inhibit the activity of anaerobic microorganisms, particularly methane-producing archaea (MPA) and simultaneously lower the reductive reactivity of the azo bond to prolong the degradation cycle [58].

By comparing the treatment performance of dyes with different structures, Turgay et al. confirmed that a methyl (-CH_3_) or methoxy (-OCH_3_) group substituted at the ortho position of the benzene ring increased steric hindrance and impeded the action of azoreductase on the azo bond [59]. Furthermore, extending the conjugated chain enhanced the chromophoric strength of the dye but simultaneously increased the electron cloud density, causing a negative shift in the reduction potential and requiring a higher reduction energy barrier for azo bond cleavage [60].

### 2.2. Anaerobic Biodegradation Pathway of Dyes

The degradation of azo dyes under anaerobic conditions is a nonspecific process dominated by biological reduction, supplemented by abiotic reduction. It begins with the reductive cleavage of the azo bond and relies on cofactors, reducing agents (such as sulfide and cysteine), and redox mediators to achieve efficient electron transfer [61,62,63]. Under anaerobic conditions, the reduction of azo bonds mainly achieves decolorization and does not necessarily result in complete mineralization. The resulting aromatic amines may persist and retain toxicity. Therefore, an aerobic or oxidative post-treatment step is often needed to promote their further transformation, detoxification, and mineralization [64,65].

**Figure 2:**
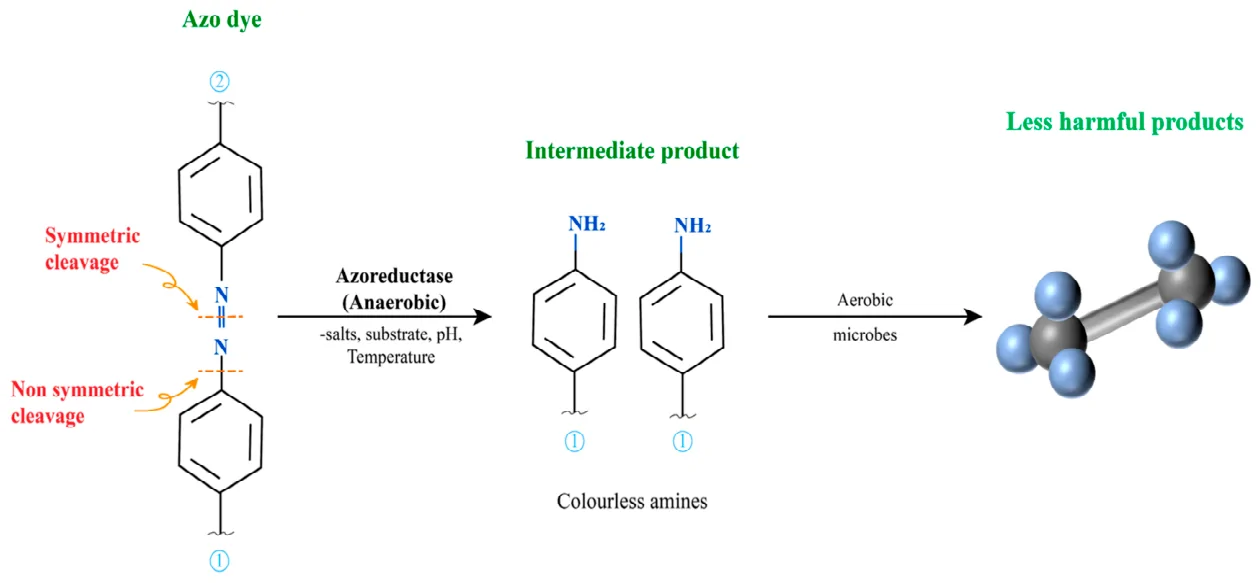
Proposed anaerobic and aerobic transformation pathways of azo dyes.

At present, the principal intracellular reducing cofactors involved in azo dye reduction include FADH_2_ and NAD(P)H [66]. Anaerobic microorganisms can utilize organic compounds such as glucose and acetate in wastewater as electron donors, generating reduced coenzymes like NADH and FADH_2_ through intracellular metabolism. Azoreductases then transfer electrons from these coenzymes to azo dye molecules, reducing the azo bond to unstable intermediates, and subsequently producing aromatic amines as intermediate products for dye decolorization [67,68]. Degradation of azo dyes by bacteria typically commences with the cleavage of the azo bond by azoreductase. The corresponding amines formed through anaerobic reduction are often poorly biodegraded under anaerobic conditions, causing their degradation rate to lag behind azo-bond reduction [69]. The mineralization of aromatic amines is more common under aerobic conditions, as shown in Figure 2. However, it has been reported that the presence of oxygen generally inhibits the azo bond reduction activity because aerobic respiration may dominate the utilization of NADH, thus impeding the electron transfer from NADH to the azo bond. Previous studies have also indicated that aromatic amines containing -OH and carboxyl (-COOH) groups can be mineralized under anaerobic conditions, and certain aromatic amines prone to autoxidation can also be degraded as well [70].

Anaerobic biodegradation of azo dyes depends on the metabolic activity of specific microbial communities. Dye stress can select microbial populations adapted to toxic conditions. In an AnMBR treating textile wastewater, the relative abundance of *Lactobacillus* increased under dye and salinity stress [71]. Organic acids produced by *Lactobacillus* may provide electron donors for other functional microorganisms. Additionally, the abundance of *Desulfovibrio* was also found to increase significantly for textile wastewater treatment by an AnMBR. By utilizing sulfate as the terminal electron acceptor, *Desulfovibrio* indirectly promoted the reduction of azo dyes and reduced toxicity accumulation [72]. The above findings can offer key insights into the degradation of azo dyes and the design of more efficient treatment strategies for dye molecules.

## 3. Factors Influencing the Effect of Azo Dyes on AnMBR Treatment Performance

In recent years, AnMBRs have been extensively studied as a technology for methane recovery from wastewater [17,18]. Specifically, AnMBR combines the advantages of anaerobic digestion (AD) with membrane filtration, achieving efficient organic matter removal and methane production [6,73,74]. The AD process comprises the stages of hydrolysis, acidogenesis, acetogenesis, and methanogenesis [75]. Meanwhile, by-products generated from this process, such as biogas and fertilizer, can enable the sustainable utilization of energy [76].

Under anaerobic biological conditions, azo dyes generally undergo reductive cleavage of the chromophoric -N=N- bond, resulting in decolorization and the formation of aromatic amine intermediates, which may require subsequent aerobic treatment for further degradation [55,77]. Previous research has demonstrated that when treating azo dyes such as Reactive Orange 16 (RO 16) and Acid Crimson GR 64 with AnMBR, the system exhibited excellent decolorization performance (generally exceeding 90%). In particular, Qiu et al. demonstrated that a halophilic/halotolerant microbial consortium achieved 93.3–99.2% decolorization of several azo dyes at 600 mg/L within 24 h under oxygen-limited conditions and retained over 90% decolorization of Direct Black G at a concentration as high as 2.4 g/L [78]. When treating real textile wastewater, the decolorization efficiency of AnMBR ranged from 53% to 77% [72]. In contrast, the decolorization efficiency of conventional aerobic MBR for azo dyes was typically below 60% and often required subsequent advanced oxidation processes for enhanced treatment [79]. For AnMBR systems treating azo dye wastewater, the primary objectives are to achieve high decolorization efficiency and COD removal, as well as stable methane production as an energy source. These treatment indicators are influenced by dye characteristics and are linked to membrane fouling development.

### 3.1. Concentration of Dyes

Dye concentration affects AnMBR treatment performance, but the response is system-specific. Electron-donor availability, microbial acclimation, dye structure, salinity, and operating conditions determine whether stable decolorization is maintained or anaerobic activity is inhibited [19,80]. For example, Spagni et al. operated a submerged anaerobic membrane bioreactor in which Reactive Orange 16 (RO16) concentrations were progressively increased from 0.060 to 3.2 g/L [9]. The reactor was initially acclimated at 60 mg/L and achieved over 90% decolorization. At 3.2 g/L, decolorization remained above 99%, whereas methane production was inhibited by approximately 80% and volatile fatty acids (VFAs) accumulated [9].

In AnMBRs, microbial activity and environmental stress can alter the production and composition of extracellular polymeric substances (EPSs). EPS mainly comprises proteins (PN) and polysaccharides (PS). Soluble EPS and soluble microbial products (SMPs) can block membrane pores, whereas bound EPS contributes to the formation of a gel and cake layer. The PS/PN ratio indicates EPS composition but cannot independently predict fouling. It should be interpreted together with EPS/SMP concentrations, microbial activity, TMP development, and flux decline [81,82,83]. A similar pattern was observed by Guo et al. when treating Acid Brilliant Scarlet GR, where the SMP concentration was only 23.75 mg/L, the tightly bound EPS (TB-EPS) concentration was 18.12 mg/g-VSS, the fouling layer structure was loose, and the TMP could be rapidly restored after physical cleaning [71].

**Figure 3:**
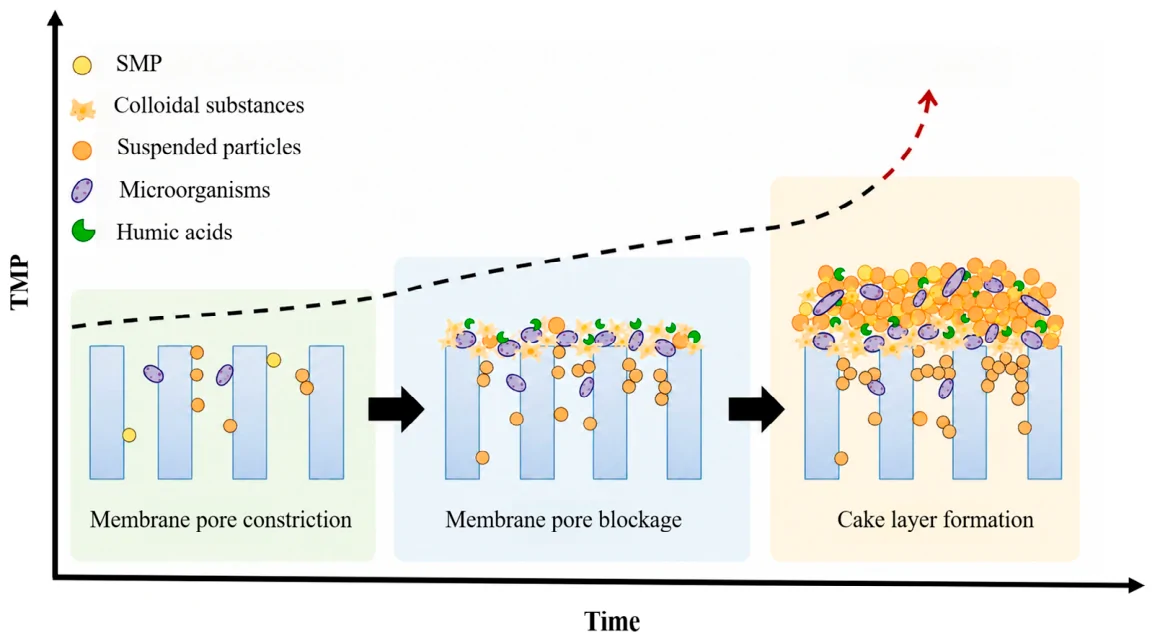
Temporal variations in TMP and membrane fouling.

At higher dye loadings, inhibitory effects on anaerobic digestion may become more pronounced. Dye reduction can compete with methanogenesis for reducing equivalents, while dyes and their transformation products may inhibit MPA, leading to volatile fatty acids (VFAs) accumulation and reduced COD removal [9,84]. Dai et al. introduced azo dyes at concentrations of 0–600 mg/L into an AnMBR and operated it continuously for 127 days [84]. As the concentration of dye increased, COD removal and decolorization declined, while VFAs accumulated. Above 450 mg/L, VFAs began to accumulate markedly, and methane production rate decreased synchronously, indicating marked deterioration of system performance. High dye loading may also induce stress-related changes in SMP and EPS production, promoting cake layer development and increasing filtration resistance [81,82,83,85]. These effects may be amplified under saline conditions. In an AnMBR treating azo dye wastewater, salinity stress increased SMPs, EPSs, and the PS/PN ratio, accompanied by more severe membrane fouling [71]. Yurtsever et al. likewise identified foulant accumulation as an important operational limitation during AnMBR treatment of real textile wastewater [72]. Figure 3 shows the evolution of TMP and membrane fouling over time. The reported concentrations should therefore be interpreted as study-specific observations rather than universal thresholds.

Operating parameters can partly mitigate the adverse effects of high dye loadings. Spagni et al. reported that a low flux of approximately 2 L/m^2^·h slowed cake-layer development in a single-dye laboratory AnMBR study [9]. This result cannot be directly extrapolated to real textile wastewater. Pilot-scale studies are needed to define practical operating ranges under mixed-dye and saline conditions.

Real textile wastewater contains salts, surfactants, starch, dispersants, and other auxiliary chemicals in addition to dyes [86]. Salinity can affect anaerobic microbial activity, SMP/EPSs secretion, and membrane fouling behavior. Guo et al. reported that increasing salinity from 0 to 5% in an AnMBR treating azo dye wastewater decreased decolorization and COD removal, increased VFAs, SMP, and EPS concentrations, and aggravated membrane fouling [71]. Similarly, Wang et al. found that increasing NaCl concentrations from 0 to 20 g/L reduced COD removal from 92% to 73% in a ceramic AnMBR treating textile wastewater. Biogas production was inhibited at NaCl concentrations above 10 g/L, although dye removal was less affected [18]. Surfactants can further modify dye organic-matter aggregation and interactions on the surface of the membrane. In textile wastewater membrane filtration, interactions among surfactants, reactive dyes, and effluent organic matter caused marked flux decline [87]. Therefore, dye loading, salinity, surfactant concentration, and other co-pollutants should be jointly considered when evaluating AnMBR fouling with real textile wastewater.

### 3.2. Structures and Their Metabolites

Differences in molecular structures of dyes also affect the performance of AnMBR. This section examines how azo-bond number, substituents, and transformation products affect dye reduction, microbial stress, and membrane fouling.

#### 3.2.1. Number of Azo Bonds

The number of azo bonds, as the core structural unit of azo dyes, directly determines the efficiency of biodegradation and the complexity of resulting metabolites. Monoazo dyes such as Methyl Orange and RO 16 possess relatively simple molecular structures, and their azo bonds are readily cleaved by azoreductase under anaerobic conditions [88,89]. The metabolic pathway of this process is relatively simple, with minimal accumulation of intermediate metabolites. Consequently, the microbial metabolic process is stable, the secretion of SMPs and EPSs is relatively low, and the membrane surface is dominated by a loose, reversible cake layer. Wang et al. treated simulated dye wastewater containing Methyl Orange and found that at a salinity of 10 g/L, the SMPs concentration peaked at 178 mg/L, primarily consisting of polysaccharides and proteins [19]. Nonetheless, as the microorganisms gradually adapted to the high-salinity environment of 20 g/L, salt-tolerant bacteria such as *Longilinea* and *Bosea* became the dominant microbial populations; the SMPs concentration decreased to 133 mg/L, and the membrane fouling rate dropped from 2.00 to 0.36 kPa/d. The decrease in the fouling rate mentioned in the experiment was observed during a long-term acclimation process. On the other hand, for unacclimated systems or under dye shock loading conditions, even monoazo dyes can still trigger a short-term peak in membrane fouling. Similarly, Amin et al. conducted comparative tests on azo dyes with different structures at various membrane fluxes and concentrations, and they found that simple, low-molecular-weight azo dyes generally exhibited higher decolorization rates, whereas the color removal of complex, high-molecular-weight dye molecules was reduced [90].

**Figure 4:**
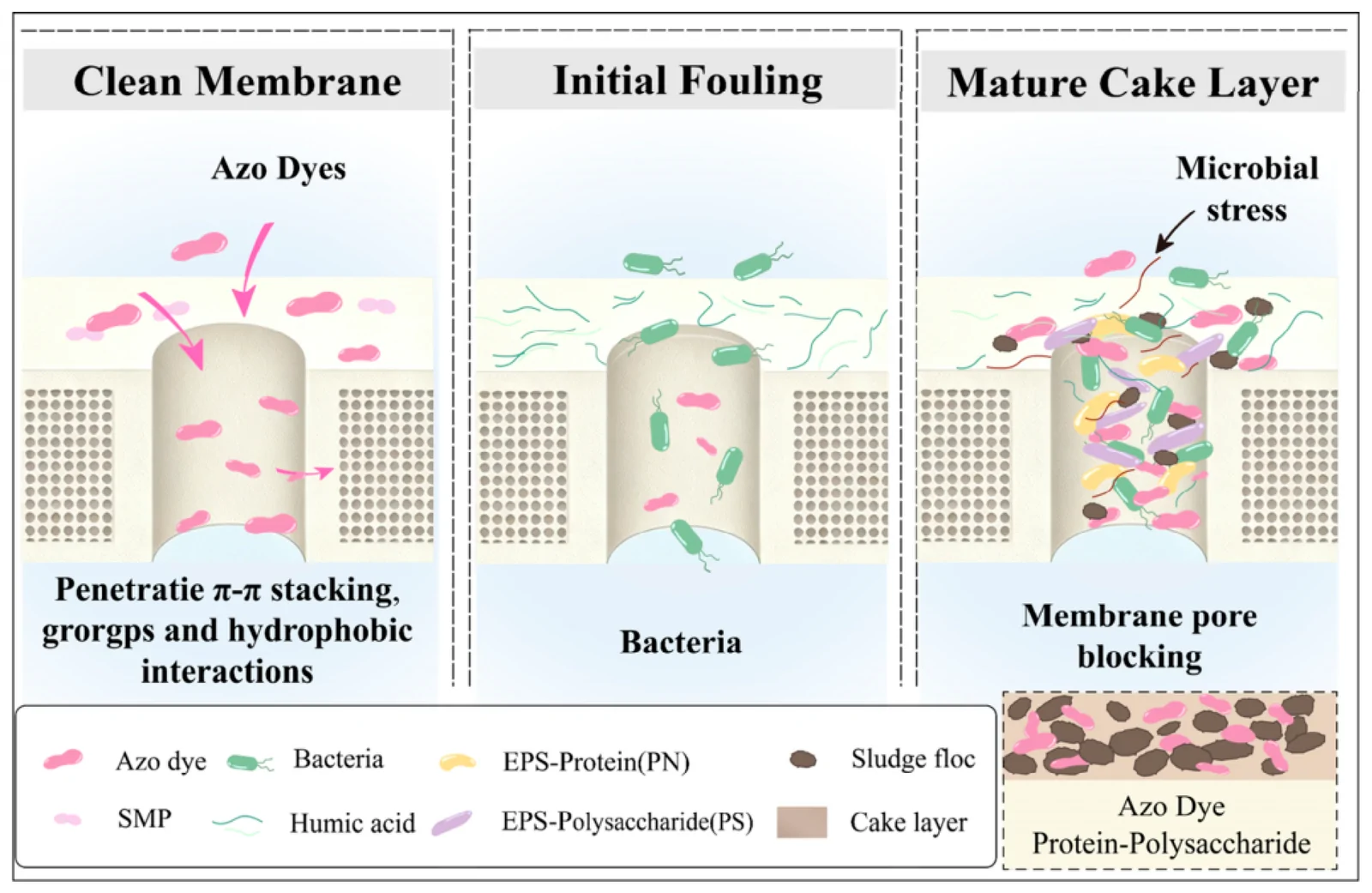
Variation in the adhesion of azo dyes and EPSs on the membrane surface.

In addition, the degradation rate of polyazo dyes is slow, and the intermediate products can inhibit microbial activity, especially that of MPA, generating cell lysis and the release of intracellular substances such as nucleic acids and proteins. Simultaneously, microorganisms secrete large amounts of EPSs to resist this persistent stress [79]. Long conjugated chains formed by aromatic rings can increase hydrophobicity and promote π-π interaction. During AnMBR treatment, this can readily trigger a surge in SMPs/EPSs secretion and exacerbate membrane fouling. When treating real textile wastewater containing Direct Black 22, it was found that the sulfide generated during the degradation process can form precipitates with metal ions present in the wastewater [79]. These precipitates, combined with EPSs, deposited on the membrane surface, bringing about membrane pore blocking and cake layer densification. The irreversible fouling accounted for 2.03% of the total fouling, and chemical cleaning with 1000 mg/L NaOCl was required to partially restore membrane performance. Figure 4 shows the enrichment process of dyes, EPS, and other substances on the membrane.

#### 3.2.2. Substituent Groups

The type and position of substituents affect the reduction reaction of anaerobic azo dyes by altering the electron distribution and steric environment around the azo bond. Electron-withdrawing substituents may facilitate electron transfer, whereas bulky substituents located near the azo bond may hinder its accessibility and retard decolorization [57]. Azo dyes containing heterocyclic rings or substituent groups exhibit unstable biodegradation performance due to the complex electron cloud distribution in their conjugated systems. Substituents such as hydrophilic sulfonic acid and hydroxyl groups can enhance the water solubility of the dyes and their adsorption affinity to the membrane surface, thereby further exacerbating membrane fouling [71]. At elevated dye concentrations, sulfonate groups may facilitate the cation bridging between EPS components, thus promoting the foulant deposition on the membrane surface. In contrast, nitro and halogen substituents mainly affect reduction kinetics. Their electron-withdrawing effects can lower the electron density at the azo bond and stabilize reduction intermediates, thus facilitating electron transfer and potentially accelerating decolorization [57,91]. Zhao et al. demonstrated that azo dyes bearing electron-withdrawing substituents close to the azo linkage underwent faster chemical and biological reductive decolorization than those containing nearby electron-donating substituents [8]. Yang et al. further demonstrated that electron-withdrawing substituents, such as sulfonate and nitro groups, generally promoted azo-bond cleavage and dye removal in bioelectrochemical systems more effectively than electron-donating groups, although the reaction kinetics were additionally governed by substituent position and steric hindrance [57]. However, substituent effects on microbial toxicity and membrane fouling cannot be inferred from decolorization kinetics alone. Aromatic-amine profiles, methanogenic activity, EPS/SMP production, and filtration resistance should be evaluated together. Rapid decolorization and difficult mineralization represented a key challenge in the treatment of highly toxic azo dyes [59]. The nature of the substituents and their position relative to the azo bond also exerted different effects on the degradation behavior and toxicity of the dyes.

#### 3.2.3. Metabolites

Aromatic amine compounds such as aniline, sulfanilic acid, and polycyclic aromatic amines, which are the products of the anaerobic reduction of azo dyes, possess the ability to intercalate into and disrupt the phospholipid bilayer of microbial cell membranes. Guo et al. further discovered that under a high-salinity (5%) environment, cell membrane damage was exacerbated, accompanied by an abnormal increase in the relative abundance of *Lactobacillus* to 49.88%, while the abundance of typical MPA sharply declined, resulting in a decrease in COD removal efficiency to 58.6% [71]. Furthermore, Turgay et al. confirmed that the aromatic amines produced during anaerobic biological treatment, after intercalating into the cell membrane, increased membrane permeability, and the mineralization efficiency of aromatic amines was significantly lower than that of azo bond reduction, with the residual fraction of aromatic amines in synthetic dye wastewater reaching 20–30% [59]. In an experiment treating the azo dye RO16 using a submerged AnMBR, Spagni et al. found that when the RO16 concentration was increased to 3.2 g/L, the system could still maintain a decolorization efficiency of over 99%, while the accumulation of VFAs reached a maximum of 1.2 g/L, and the methane yield decreased by 80–85% [9]. This demonstrates both the high efficiency of anaerobic biological reduction in cleaving the azo bond and the toxic inhibition of the methanogenic community by aromatic amines. As recalcitrant organic compounds, the incompletely degraded aromatic amines can directly affect effluent COD, preventing it from meeting discharge standards, thus necessitating their mineralization through subsequent aerobic processes or advanced treatment technologies.

Hydrophobic aromatic amines themselves can also adsorb and deposit on the membrane surface through van der Waals forces and form an irreversible fouling layer [92]. More importantly, the toxic inhibition caused by aromatic amines can lead to the accumulation of acetic and propionic acid among diverse VFAs [93]. VFA accumulation can reduce the pH, thus promoting the protonation of charged groups such as carboxyl and phosphate groups in EPSs [94]. This weakened the hydrophilicity of EPSs and enhanced their adhesion strength to the membrane surface. Simultaneously, the high concentration of VFAs altered the microbial community structure, disrupted the AD balance, and indirectly increased sludge viscosity and cake layer compactness [95].

Overall, rapid decolorization does not necessarily indicate stable AnMBR performance. Dye structure, aromatic-amine toxicity, VFA accumulation, microbial inhibition, and EPS-related fouling should be evaluated together with decolorization efficiency.

### 3.3. Microbial and Electron-Transfer Factors

Dye structure and transformation products can alter microbial activity and electron-transfer pathways. These changes affect azo-dye reduction, VFA conversion, EPS/SMP production, and the subsequent development of membrane fouling. Sulfate-reducing bacteria (SRB) can play an important role in the engineering of anaerobic azo dye degradation. SRB can produce azoreductases that catalyze azo bond cleavage and can also use SO_4_^2−^ as a terminal electron acceptor to facilitate reduction [96]. Prato-Garcia et al. reported that the addition of anthraquinone-2,6-disulfonate (AQDS) enhanced chemical- and biological-sulfide-assisted azo-dye decolorization. In the absence of AQDS, biological sulfide was more efficient than chemical sulfide in removing azo dyes [61]. In addition, Yurtsever et al. confirmed the decolorization efficiency of Remazol Brilliant Violet 5R dye in the anaerobic stage was close to 100% via a sequential AnMBR-AeMBR system [97]. This process was dominated by SRB, which can utilize dye molecules as electron acceptors and achieve azo bond cleavage in the presence of sulfate. Recent evidence suggests that anaerobic azo-dye decolorization involves both enzyme-associated reduction and extracellular electron transfer rather than the direct action of secreted reductases alone [98]. Conductive materials can enhance electron transfer to the azo bond and regulate VFA conversion [99]. Their benefits should be evaluated together with their effects on microbial selection, membrane fouling, material stability, and cost.

## 4. Mitigation Strategies

Fouling in AnMBRs treating dye wastewater is influenced by membrane flux, sludge characteristics, related dye foulants and membrane properties [100]. Membrane fouling contributes to an increase in TMP and a decline in membrane flux, while the frequency of membrane cleaning and replacement compromises the economic viability of AnMBR and the growth of microbial communities [101]. In recent years, methods such as dye structure modification, membrane material modification, and chemical cleaning have been progressively applied to the treatment of azo dye wastewater, which can enhance system stability and mitigate membrane fouling.

### 4.1. Modification of Dye Structure

In response to the issues of slow degradation of polyazo dyes and high toxicity of aromatic amines identified in Section 2.1, researchers have optimized dye structures through molecular design to reduce the difficulty of biodegradation at the source. Yang et al. demonstrated that molecular structure can determine degradation kinetics by influencing the electron density and steric hindrance of the azo bond through experiments and quantitative structure-activity relationship (QSAR) model fitting [57]. Therefore, under the premise of ensuring dyeing performance, molecular modification may enhance dye biodegradability and reduce the adsorption capacity and biotoxicity of microbial metabolites by modulating the steric hindrance and hydrophilicity/hydrophobicity of dye molecules through molecular modification.

The number of azo bonds is positively correlated with the difficulty of degradation. Comparative experiments in a carbon-based AnMBR operated at 37 ± 1 °C showed that the monoazo dye Acid Orange 7 (AO7) was more readily decolorized than the structurally more complex diazo and triazo dyes Reactive Black 5 (RB5) and Direct Blue 71 (DB71). Each dye was tested at 50–100 mg/L with sodium acetate as a co-substrate (dye/acetate mass ratio of 1:3) and a permeate flux of 0.05–0.10 L/m^2^·h. At 50 mg/L and 0.05 L/m^2^·h, decolorization reached 98%, 82%, and 72% for AO7, RB5, and DB71, respectively [90]. Furthermore, maintaining a linear conjugated chain structure in molecular design can effectively reduce the chemical stability of the molecule, making the azo bond more susceptible to cleavage by the reductases of anaerobic microorganisms. When introducing heterocycles such as pyrazole and thiazole, it is also necessary to control the conjugation extension between the heterocycle and azo bond to avoid the formation of an excessively long conjugated system.

Atay et al. successfully synthesized new monoazo dyes using a pyrazole heterocyclic skeleton [102]. Elgammal et al. directionally introduced hydrazide-hydrazone (-CONH-N=CH-) groups into dye molecules and evaluated their dyeing, antimicrobial, antibiofilm, and molecular-docking properties [103]. Such structural modifications can redistribute electron density and alter molecular conformation. They can also change the predicted interactions between dyes and defined microbial protein targets. Selected dyes showed antibacterial activity against *Staphylococcus aureus* and *Bacillus subtilis*. Selected dyes also inhibited *S. aureus* biofilm formation [103]. These biological activities do not alone demonstrate reduced membrane biofouling. Such performance should be confirmed by microbial-attachment, EPS, filtration-resistance, flux-recovery, leaching, and ageing tests under relevant wastewater conditions [104,105,106,107]. Dye biodegradability is a separate property. It depends on azo-bond accessibility, redox reactivity, substituent effects, and steric hindrance [57]. Antimicrobial activity may affect the viability or activity of dye-degrading microorganisms. Biodegradation, mineralization, aromatic-amine analysis, and microbial-community tests are therefore required before this strategy can be considered environmentally viable [108].

### 4.2. Modification of Membrane Modules

**Figure 5:**
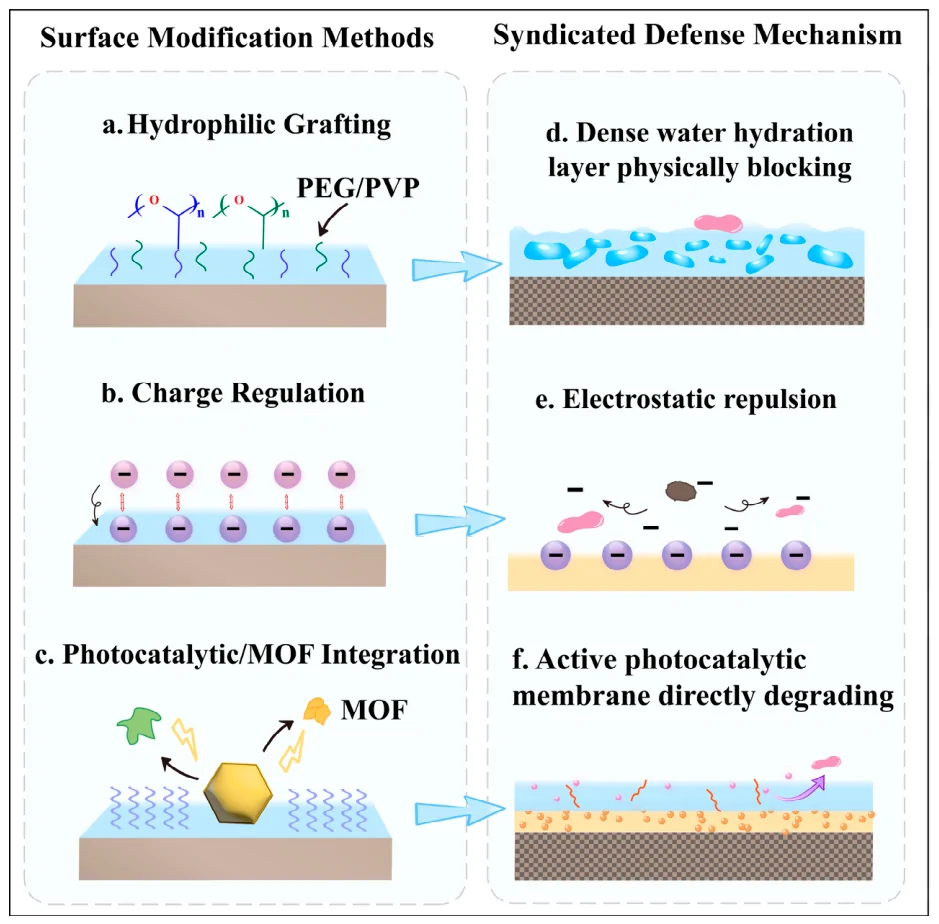
Surface modification strategies for membrane optimization.

In dye wastewater, adsorption-driven membrane fouling depends on the molecular structure and chemical speciation of the dyes and their transformation products, as well as on pH, ionic strength, and membrane chemistry. Hydrophobic or aromatic dye-related compounds, including some azo dyes and aromatic amine metabolites, may adsorb onto hydrophobic membrane domains [109]. Aromatic moieties on the membrane or coating may promote adsorption through π-π interactions [109,110]. Dye exposure may stimulate EPS-related biosynthesis in certain microorganisms [111]. EPS accumulation and spatial distribution have been associated with compact membrane biofilms, cake layer development, and increased filtration resistance [112,113]. As illustrated in Figure 5, membrane-surface modification may reduce organic-foulant attachment and initial microbial adhesion by reducing surface roughness, increasing hydrophilicity, and tailoring surface charge [114]. A stable antimicrobial function is required when microbial inactivation is the objective.

AnMBRs commonly employ hydrophobic membranes, such as polyvinylidene fluoride (PVDF) and polytetrafluoroethylene (PTFE), for wastewater treatment [115,116,117]. Hydrophobic or aromatic dye-related compounds may adsorb onto hydrophobic membrane domains, whereas many sulfonated azo dyes remain water-soluble. Hydrophilic surface modification can lower the water contact angle and reduce foulant adhesion. Surface charge can also influence the adsorption of ionic dyes. Negatively charged functional groups may repel anionic dyes under suitable solution conditions, thus reducing pore adsorption and delaying the formation of the cake layer.

Zheng et al. modified a PVDF membrane with Cu-MOF-74, improving hydrophilicity and reducing surface roughness [118]. Wang et al. applied a surface-conductive hollow-fiber membrane in an AnMBR. The surface-electric-field AnMBR (S-AnEMBR) enhanced organic matter degradation and enriched alkaliphilic MPA at the cathode by 48%. Meanwhile, the PS/PN ratio of loosely bound EPS (LB-EPS) in the S-AnEMBR was 3.82, which was 21.00% lower than that in the control AnMBR [119]. These results suggest that conductive membrane surfaces can influence microbial activity and EPS production [120].

Xiao et al. reported that poly heptazine imide (PHI) modification increased membrane hydrophilicity and reduced the contact angle of PVDF from 77.86° [121]. The negative surface charge of PHI may increase electrostatic repulsion toward anionic dyes. Fan et al. prepared a TpPa-SO_3_H/PVDF membrane containing -SO_3_H and -OH groups, which exhibited high hydrophilicity [122]. The water contact angle decreased substantially from 114° to 50.8°, and the lower surface roughness may alleviate foulant adhesion. The modified membrane achieved photocatalytic degradation efficiencies above 90% for several dyes. An et al. prepared an NH_2_-MIL-125 modified membrane-distillation system that maintained a flux of 41.29 L/m^2^·h in dye wastewater containing high salinity [123]. Its photocatalytic activity reduced dye-induced fouling. However, adsorption of Congo Red decreased degradation efficiency and accelerated wetting. Vatanpour et al. prepared a PVDF membrane with a zeolitic imidazolate framework-8 layer, which improved hydrophilicity, membrane flux and the removal rate of azo dyes [124].

Membrane pore size is another important design variable. Microfiltration (MF) membranes generally provide higher permeability, while ultrafiltration (UF) membranes provide greater retention of particulates and macromolecules [21]. Pore size can also influence fouling mechanisms. In MBR studies, small pores were associated with protein-like SMP capture and irreversible internal pore blocking, whereas larger pores favored surface cake-layer formation [125,126]. Because most dissolved azo dyes are smaller than typical MF and UF pores, dye removal in AnMBRs mainly depends on bioreduction, adsorption, and dynamic-cake-layer retention rather than direct size exclusion [21,127]. Therefore, pore size should be selected together with membrane material, flux, wastewater composition, and the fouling control strategy.

### 4.3. Chemical Cleaning and Bioaugmentation

Although some membrane modification methods have significantly improved the antifouling capability and dye rejection efficiency of AnMBRs when treating wastewater containing azo dyes, membrane pore blockage and irreversible fouling caused by EPSs and intermediate degradation products may still be unavoidable during long-term operation under high loading conditions. Periodic relaxation and air scouring can limit the accumulation of loosely attached EPS-rich cake layers through shear-induced foulant removal [37]. Backwashing can remove reversible foulants, but its effect on fouling-layer structure depends on membrane configuration, backwash flux, duration, and foulant composition. Excessive backwash intensity may redistribute foulants, promote pore intrusion, or compromise membrane integrity and hydraulic performance [128]. Therefore, backwashing should be optimized and evaluated using TMP development, permeability recovery, and membrane-integrity testing rather than flux recovery alone [94].

However, offline chemical cleaning may cause membrane damage, increase operating costs, and generate secondary waste. Park et al. found that osmotic backwashing improved water flux by 10.8% compared to pure chemical cleaning and was capable of removing foulants close to the membrane surface from natural organic matter [129]. Furthermore, Fan et al. achieved in situ membrane cleaning through an indirect high-voltage electric field, which reduced the TMP by 66.7% compared to a conventional AnMBR without affecting AD performance [130]. Meanwhile, with increased cleaning frequency, when *Brooklawnia* and *Propioniciclava* were inhibited, fermentative bacteria such as *Syntrophomonas*, *Synergistaceae*, and *Fermentimonas* were enriched as a compensatory metabolic mechanism. This shift in the microbial community improved COD removal efficiency and methane production.

In terms of bioaugmentation, Li et al. demonstrated that dual bioaugmentation extended the time to membrane fouling by up to twofold and increased cumulative methane production by 18% relative to the control [131]. In addition, Patiu et al. developed an ultrasonic atomization membrane system, in which the graphene oxide layer was modified with formaldehyde to enhance hydrophilicity and surface charge, while ultrasound ensured its high pure water flux (129 ± 13 L/m^2^·h). The experimental results showed that the system achieved 90% rejection for Methyl Orange and 99% rejection for both Congo Red and Disperse Blue 1 [132]. Overall, these experiments demonstrate that chemically enhanced cleaning can mitigate membrane fouling effectively, whereas bioaugmentation with verified quorum-quenching or EPS-modulating strains may improve antifouling performance by altering EPS production and microbial-community composition [128,133]. During membrane operation, verified functional strains may be applied as a preventive measure to reduce EPS production. Maintenance chemical cleaning can then be performed when TMP reaches a site-specific threshold [133,134,135]. In a full-scale municipal MBR, the annual cost of cleaning chemicals was estimated at 14.02 million SEK under a scheduled sodium-hypochlorite/citric-acid cleaning regime and 6.26 million SEK under demand-driven cleaning [136]. Although these strategies have shown promising results under laboratory conditions, their implementation at engineering scale faces technical and economic limitations.

For instance, hydrophilic coatings and MOF-based membrane modifications may have insufficient long-term durability. Their adhesion, chemical stability, and resistance to mechanical wear or material leaching should be verified under continuous operation and cleaning conditions [137]. Electrical cleaning methods such as conductive membrane modules and in situ electric-field cleaning can achieve real-time control of membrane fouling. However, scale-up requires investment in conductive materials, electrodes, power supplies, and maintenance. Reported specific energy consumption for electrochemical membrane technologies ranges from 0.01 to 5 kWh·m^−3^ depending on the membrane material, feedwater characteristics, and treatment configuration [138,139]. Although bioaugmentation is relatively simple to implement and avoids additional chemical dosing, its antifouling efficacy depends on the persistence and activity of the introduced functional strains under variable operating conditions and interactions with native microbial communities [133,134].

No individual strategy has demonstrated durable and cost-effective fouling control in real textile wastewater. Membrane modification may reduce foulant attachment but requires long-term evaluation of coating durability and leaching. Cleaning can restore permeability but increases chemical use, energy consumption, or membrane-aging risks. Bioaugmentation may reduce EPS production, but its long-term efficacy depends on the persistence of introduced strains.

## 5. Conclusions and Future Perspectives

### 5.1. Conclusions

This review systematically elucidates the interplay between azo dye molecular structures and the performance of AnMBRs. Through analysis of molecular structural characteristics, AD pathways, and microbial community responses, primary conclusions are listed below:

1. Monoazo dyes with simpler conjugated systems undergo rapid enzymatic cleavage. In contrast, the degradation of polyazo dyes is hindered by higher reduction energy barriers, yielding recalcitrant polycyclic aromatic amines. Electron-withdrawing substituents (e.g., –SO_3_H, –NO_2_) may promote initial decolorization depending on their position relative to the azo bond and the associated steric hindrance.
2. Accumulation of hydrophobic aromatic amines disrupts microbial cell membrane integrity, inducing metabolic stress that stimulates excessive secretion of SMPs and EPSs, particularly increasing the PS/PN ratio. Combined with VFA accumulation, these foulants densify the cake layer and aggravate irreversible fouling on conventional hydrophobic membranes (e.g., PVDF and PTFE).
3. Current research has explored various effective strategies: (i) membrane surface modification by introducing hydrophilic groups and negatively charged functional groups, utilizing steric hindrance and electrostatic repulsion to resist dye adsorption; (ii) the introduction of novel nanomaterials or surface conductive membranes to promote in situ oxidation of organic matter; and (iii) the adoption of physical methods such as osmotic backwashing and in situ electro-cleaning, or the combination of bioaugmentation and chemical cleaning to prolong the replacement cycle of the membrane.

### 5.2. Future Perspectives

Based on the above analysis, future perspectives are as follows:

1. The biotoxicity, transformation products, and membrane fouling effects of newly designed dyes require further investigation.
2. Membrane fouling control mainly relies on membrane modification and operational optimization. Data-driven models may integrate influent dye concentration, salinity, pH, organic loading rate, flux, TMP, EPS/SMP characteristics, and methane-production indicators to identify early fouling risks. Future studies should validate these models using independent long-term datasets and real textile wastewater before applying them to cleaning decisions.
3. Most studies use synthetic wastewater and may not capture the combined effects of coexisting constituents in real wastewater. Future work should assess the long-term stability and operational boundaries of AnMBRs treating real textile wastewater containing diverse dye structures and co-pollutants.

## Figures and Tables

**Table 1:** Decolorization efficiencies of selected dyes in biological reactors.

Azo Dye	Bioreactor	Inf. COD (mg/L)	COD Removal (%)	Decolorization (%)	HRT (h)	Ref.
Acid Orange 7	MBBR	1000	20.6 ± 3.6	36.7	12	[26]
Acid Orange 7	UASB	2000	75.2	79.9	12	[27]
Reactive Red 2	SBR	400	73.58	54.03	12	[28]
Reactive Black 5	UASB	125	38	75	11.5	[29]
Reactive Black 5	SBR	1050	70.4	83	12	[30]
Reactive Red X-3B	Microbial fuel cell	500	60.1	85.2	24	[31]
Methyl Orange	UASB	192	-	94.83	36	[32]
Reactive Black	Anaerobic baffled reactor	305	42	45	18.6	[33]
Direct Red 28	Up-flow immobilized packed bed bioreactor	1300	-	93	85	[34]
Direct Red 75	MBBR	2347	67.9	85.00	48	[35]
Direct Black 22	SBR	1200	76.4	81.4	24	[36]

**Table 2:** Structural characteristics and relevant properties of representative synthetic dye classes.

Types	Main Chromophore Structure	Key Properties	Typical Dyes	Ref.
Azo dyes	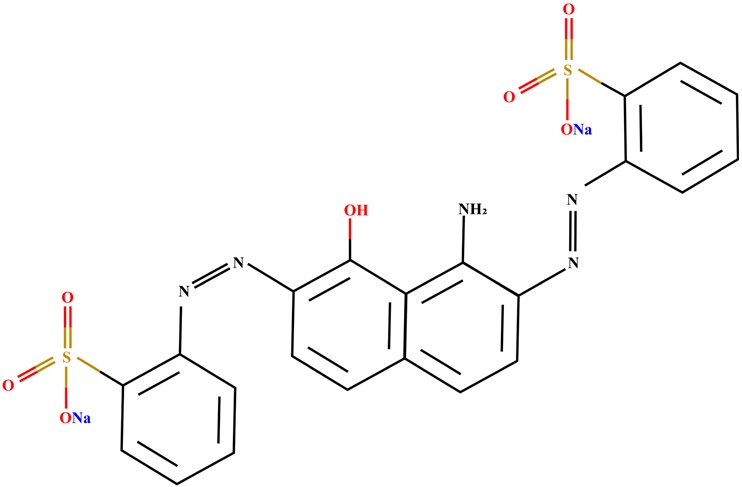	Often water-soluble when sulfonated, undergo reductive azo-bond cleavage under anaerobic conditions; aerobic persistence varies with structure	Azo red 2, Procion red-H3B, Remazol red R, Acid blue 29	[41,42,43,44]
Anthraquinone dyes	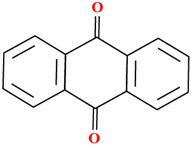	High chemical and photostability, generally resistant to biodegradation	Reactive blue 19	[45,46,47]
Indigo dyes	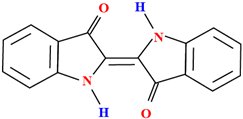	Low water solubility and bioavailability, prone to oxidation and precipitation	Indigo blue, Indigo carmine	[48,49]
Sulfur dyes	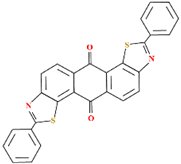	Typically have high molecular weight, limited water solubility, poorly defined structures, and sulfur-containing groups	Sulfur black 1, Sulfur black 11	[50,51]

## Data Availability

No new data were created or analyzed in this study. Data sharing is not applicable to this article.
